# Research advances of femtosecond laser-induced nanogratings for transparent materials

**DOI:** 10.3389/fchem.2022.1082651

**Published:** 2022-11-16

**Authors:** Yue Lu, Yunfei Li, Xiaofan Xie, Ziqi Tang, Lifang Li, Jiawei Li, Yu Ding

**Affiliations:** ^1^ Center for Advanced Laser Technology, Hebei University of Technology, Tianjin, China; ^2^ Hebei Key Laboratory of Advanced Laser Technology and Equipment, Tianjin, China; ^3^ Tianjin Key Laboratory of Electronic Materials and Devices, Tianjin, China; ^4^ National Demonstration Center for Experimental (Electronic and Communication Engineering) Education, Hebei University of Technology, Tianjin, China; ^5^ Science and Technology on Electro-Optical Information Security Control Laboratory, Tianjin, China

**Keywords:** femtosecond laser, nanogratings, optical storage, birefringence, transparent materials

## Abstract

Femtosecond laser-induced nanogratings generation inside transparent materials has been the focus of research in the field of femtosecond laser precision processing. Due to the advantages of optical birefringence phenomenon, periodicity, thermal stability, controllability of delay value and optical axis direction, and re-writability, nanogratings are widely used in research fields such as optical storage and optical devices. This mini-review explores the existing mechanism of femtosecond laser-induced formation of nanogratings and the progress of inducing nanogratings in different types of glass and sapphire crystals. It also describes the prospects of nanogratings for a wide range of applications in optical components and optical devices.

## Introduction

Micro and nano processing is an important part of advanced manufacturing and is a symbol of the level of high-end manufacturing (Sugioka, 2019). The emergence of femtosecond laser processing technology has brought revolutionary changes into the field of micro and nano processing (Zheng et al., 2020; Mastellone et al., 2020; Xie et al., 2021; Yan et al., 2021; Zhang et al., 2022; He et al., 2022). Femtosecond laser has a very narrow pulse width and high peak power, and the energy interacts with the material in a very short period of time during processing (Chichkov et al., 1996; Meng et al., 2019; Hua et al., 2022). Due to its non-linear absorption characteristics, it can achieve true three-dimensional processing at the focal point with high-precision (Khuat et al., 2014; Li et al., 2020). Femtosecond laser ablation can be used to prepare micro and nano structures on the surface of metals (Davydov and Antonov, 2017), semiconductors (Ionin et al., 2012; Li et al., 2020), ceramics (Perrie et al., 2005) and other materials (Gui et al., 2004; Burghoff et al., 2006; Lin et al., 2015), demonstrating its excellent micro processing capability. In addition to surface ablation, femtosecond lasers are also popular in performing microfabrication inside transparent materials (Osellame et al., 2007). In the late twentieth century, researchers studied the structural changes inside transparent materials irradiated by femtosecond lasers (Jia et al., 2020). At low pulse energies, it can write optical waveguides inside transparent materials. While at higher pulse energies, it can induce small holes or cracks. Otherwise, when the energy is in between, an ordered structure with a subwavelength periodic distribution of refractive index, which is called nanogratings, appears in the radiation region (Champion et al., 2013).

In recent years, research on femtosecond laser-induced nanogratings has focused on various types of glass materials (fused silica glasses, borate silicate glasses, etc.) and some transparent crystalline materials (mainly sapphire) (Hnatovsky et al., 2005). The birefringence effect, thermal stability, delay value and optical axis controllability, and rewritable properties of periodic nanogratings induced inside transparent materials have led to a wide range of applications in multidimensional optical data storage and polarization optics devices (Zhang et al., 2019a). In the field of polarized optical devices, polarization conversion devices and geometric phase devices can be well prepared. In summary, research on femtosecond laser-induced nanogratings inside transparent materials is imperative.

In this mini-review, we summarize the recent research progress of femtosecond lasers to induce nanogratings in several typical transparent materials, including quartz glass, GeO_2_ glass, borosilicate glass, highly porous glass and other glass materials, as well as sapphire crystals. The application of nanograting structure in optical data storage and polarized optical devices is also described. Finally, we look forward to the challenges and prospects of femtosecond lasers in inducing nanogratings in transparent materials.

## Femtosecond laser-induced nanogratings in glass

In 1994, Professor Hiroaki Misawa of Hokkaido University in Japan first observed the refractive index changes in glasses induced by ultrashort pulses. In 1996, Glezer et al. (1996) repeated the experiment of femtosecond laser-induced refractive index change inside glass with the above method; then a slurry of research was poured into this field. In 2000, Qiu et al. (2000) discovered anisotropic light scattering along the laser polarization plane in fluoroaluminate glass. At this time, some researchers (Mills et al., 2002), including Qiu, speculated that the refractive index change might be due to the creation of a nanograting structure in the vertical polarization direction in the femtosecond laser irradiation region.

Shimotsuma et al. (2003) used a backscattering electron microscope to observe the nanograting structure in quartz glass for the first time, which was finally confirmed by the scientific community. They found that nano-stripes in the laser-irradiated region were perpendicular to the laser polarization direction and only 20 nm wide, and a clear periodic distribution of oxygen defects in the dark stripes of nanogratings was revealed by the elemental detection in the irradiated region, which may be related to the formation of the nano-grating (Shimotsuma et al., 2003).

The precise control of the nanograting structure has been a hot topic of research. In 2016 Yan et al. (2016) fabricated good-quality parallel gratings on quartz glass with 1 μm adjacent line spacing and 500 lines/mm frequency. In 2019, Zhang et al. (2019c) shaped a Gaussian beam at 1,030 nm into a Bessel beam and used it to write a vacuum structure in quartz glass with Bragg grating. By controlling the energy of the Bessel beam, they precisely controlled the gap of the nano-grating below 100 nm, and realized the precise regulation of the nano-grating structure.

In addition to quartz glass, femtosecond laser-induced nanogratings have been demonstrated in a variety of glasses, including GeO_2_ glasses, borosilicate glasses, Barium-Germanium-Gallate (BGG) glasses and highly porous glasses (Shimotsuma, 2014). The nanograting structures obtained by femtosecond laser processing in GeO_2_ glasses are slightly different from those in quartz glasses. Asai et al. (2015) found that the femtosecond laser was able to induce two different structural changes in GeO_2_ glasses, morphological birefringence and dissociation. When the laser pulse energy is less than 0.2 μJ, the periodic structure consists mainly of oxygen defects, and the nanograting birefringence effect formed is greater due to the high density of nanopores in GeO_2_ glass relative to quartz glass at this time. And when the laser energy is greater than 0.4 μJ, the glass structure dissociates and generates O_2_ molecules (Asai et al., 2015).

Similarly, nanograting structures can be prepared in borosilicate glass using femtosecond laser irradiation (Zimmermann et al., 2014). Compared with nanogratings in quartz glass, borosilicate glasses exhibit smaller delays due to the reannealing of gas pores. And for the first time, nano-gratings with a period down to 60 nm were observed.

Similar to quartz glass, researchers are interested in the parameters that affect the phase shift in the birefringent region of nanogratings. Lotarev et al. (2019) used a femtosecond laser to irradiate borosilicate glass with compositions in the sub-stable liquefaction range to produce nanogranules and found that the phase shift in the birefringent region increased with the number of pulses written. Moreover, the formation of nanogratings is accompanied by the migration of sodium ions in the laser-modified region, which indicates that the parameters of nanogratings are also related to the glass composition (Lotarev et al., 2019). In 2018, Wang et al. (2018) found that the period of nanogratings prepared in borosilicate glass varies with the laser polarization direction and was related to the effect of the pulse front tilt angle. It was also found that the morphology and birefringence signal intensity of nanogratings are significantly correlated with the content of sodium oxide (Wang et al., 2018).

In 2020, Fedotov et al. (2021a) continued to study the correlation between the optical phase shift and the chemical composition of glass in the region of induced birefringence in borosilicate glasses. It was found that if the Na_2_O content in the glass was high, then a very small number of pulses could induce the production of polarized birefringent bands. As a result, the number of pulses required decreased with increasing Na_2_O content. The phase shift pseudo color diagrams of light passing through the birefringent region were then characterized for different contents and pulse durations. This finding is significant for the study of femtosecond laser-induced nanograting structures in borate silicate glasses (Fedotov et al., 2021a).

However, borosilicate glass can only realize the application of photons in the short infrared wavelength range. To overcome this limitation, scientists began working on BGG glass. Yao et al. induced the formation of nanogratings in BGG glass by femtosecond laser direct writing, and obtained a higher structural birefringence value than silicon glass and GeO_2_ glass. They also improved the stability of BGG glass by introducing transition metals (Zn). Finally, the birefringence value of −0.029 is reached in the 8% ZnO-BGG glass (Yao et al., 2022).

## Femtosecond laser-induced nanogratings in sapphire

Sapphire crystal is a transparent material with excellent performance, which is widely used in infrared window, LED device substrate and optoelectronic fields (Liu et al., 2022). The study of sapphire nanogratings dates back to 2008, when Gottmann et al. (2008) used femtosecond laser modification combined with chemical etching in aqueous hydrofluoric acid solution to fabricate deep micro-nanostructures with submicron resolution in sapphire volumes. Subsequent studies on sapphire nanogratings have proliferated.

**FIGURE 1:**
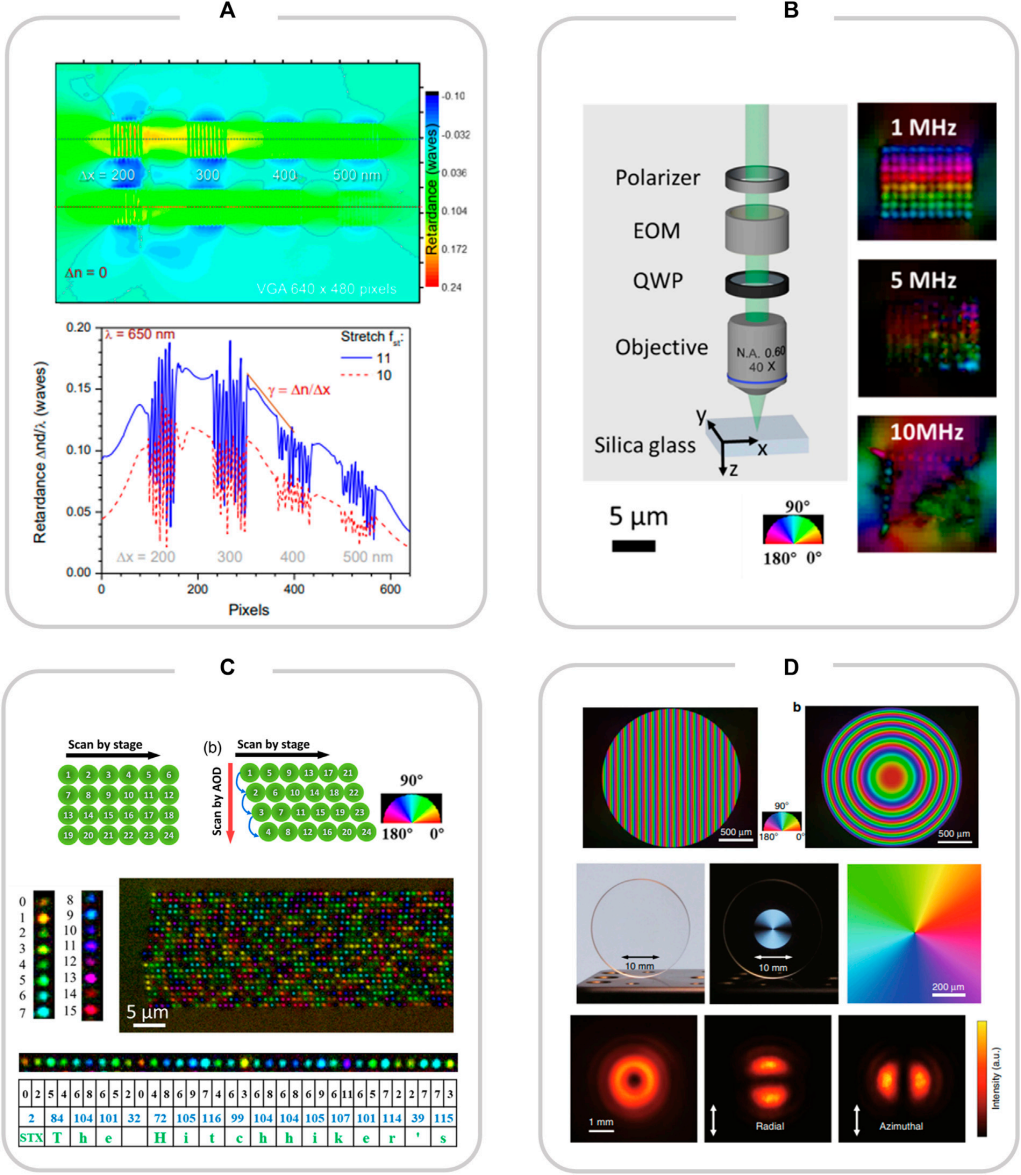
**(A)** Changes in birefringence optical resolution formed by large nanograting inside sapphire; reproduced with permission from Fan et al. (2019). **(B)** Laser-induced birefringence effect inside silica glass; reproduced with permission from Lei et al. (2021). **(C)** Femtosecond laser-induced application of silica glass nanograting in optical storage: Optical data storage for 5G data; reproduced with permission from Lei et al. (2021). **(D)** Femtosecond laser-induced nanograting applications in optics: GP prisms, GP lens and vector beam converters based on low-loss birefringence; reproduced with permission from Sakakura et al. (2020).

In 2012, Bai et al. (2012) induced nanograting planes with an average period of 230 nm inside sapphire. The direction of this nanogratings is perpendicular to the laser polarization direction, and in 2019, Fan et al. (2019) also found nanogratings with a period of 300 nm at the end of the laser incidence direction during scribing inside sapphire using an axially stretched Bessel femtosecond laser. As shown in Figure 1A, nanogratings with a period of 150–300 nm were observed at the focal point.

As with glass materials, the study of factors influencing the internal nanograting structure of sapphire is also a popular topic. In 2021, Zhai et al. (2021) found that the overall structure of the nanogratings shrank and narrowed as the scanning speed increased, and individual nano cracks with widths below 100 nm were prepared after acid etching. As the laser fluence increased, nanogratings expanded and increased in number, and the period varied between 320 and 398 nm. In addition, they achieved the erasure and rewriting of nanogratings by using two orthogonally polarized femtosecond laser pulses. This study provides new evidence for the physical mechanism of laser-induced nanogratings and paves the way for the preparation of sapphire substrate nanodevices (Zhai et al., 2021).

In addition to sapphire, nanogratings can also be induced in tellurium dioxide crystal (TeO_2_). Shimotsuma et al. used femtosecond laser to irradiate TeO_2_ crystal and formed a periodic nanograting structure inside it, with 30 nm width. TeO_2_ crystal has a high refractive index and excellent acousto-optic performance, which can be widely used in acousto-optic devices and photonic crystals. Therefore, it is also necessary to study the nanogratings inside TeO_2_ crystal (Shimotsuma et al., 2005).

## Applications

### Optical data storage

Conventional data storage has only three coordinates: X, Y and Z. In contrast, nano-grating-based data storage can provide two additional storage dimensions on the basis of traditional 3D data storage: slow axis direction and optical travel delay. This is why it is also called five-dimensional (5D) data storage. Due to the erasable nature of nano-grating, the stored optical data can also be rewritten. In addition to these advantages, the high temperature and pressure resistance and chemical resistance of glass and sapphire materials enable the nanogratings processed by femtosecond laser to have permanent optical storage capability in harsh environments. Therefore, it is a promising work to study the data storage function of nanogratings. In 2008, Taylor demonstrated the superiority of nanograting storage by using the phenomenon of refractive index change in the nanogratings to store and erase information. They successfully wrote the letter string “SIMS” and “IMS” inside the transparent material. The “IMS” letter string was obtained by irradiating the written “S” letter with a laser polarization change and then erasing it (Taylor et al., 2008).

Another advantage of nanograting data storage is 5D image storage, which has been studied with considerable results. In 2010, Shimotsuma et al. (2010) demonstrated the first 5D image storage based on nanogratings, and a 3.4 × 1.8 mm map of the world was directly written in quartz glass by a femtosecond laser. The technology is capable of storage capacity of 300 Gbit/cm^3^, which is 10 times larger than usual disks. In 2012, Beresna et al. (2012) used a femtosecond laser to write two image data, Newtonian and Maxwell, in quartz glass to achieve 5D storage. The direction of the rotating optical axis was the fourth dimension and the optical travel delay was the fifth dimension and the amount of optical travel delay exceeded 100 nm.

In addition, nano-grating-based data storage has a much longer lifetime than traditional data storage methods, allowing for almost permanent storage. Hitachi Corporation achieved permanent optical storage of data in glass for hundreds of million years with a storage density exceeding the recording density of CDs, and brought this technology to practical applications (Kovacs, 1994).

Zhang et al. (2014) demonstrated that preparing nano-grating structures can store near-infinite lifetime data inside quartz glass with a much higher data recording speed. They modulated 4 polarization directions and 2 optical range delays after hitting multiple groups of points at the same depth, ranging from 1–100 points each, which were encoded into 3bit/pc binary data. The information was then decoded for three birefringent layers 20 μm apart, resulting in only 42 errors out of the 11,664 bits of information recorded in the three layers, which could be eliminated by an additional correction procedure (Zhang et al., 2014).

In addition to the well-known advantages of nano-grating-based data storage, the structural form of the grating can also be controlled to pursue high-density, multi-level and ultra-high-speed data storage. Sakakura et al. (2020) wrote controllable birefringent structures with up to 99% transmittance in the visible and near-infrared bands directly inside quartz glass; while this spatially selective birefringent structure writing technique with high transparency also enabled high-density multilayer data storage.

In 2021, Lei et al. (2021) proposed a method to write anisotropic nanostructures in quartz glass quickly and efficiently, first generating isotropic nanocavities when the pulse energy was above the microburst threshold, and then elongating them into anisotropic nanocrystalline structures by the near-field enhancement effect of low-energy pulses, minimizing possible thermal effects (Figure 1B). The anisotropic nanostructures can be used for 5D data storage at a storage rate of 10^6^ voxels/s, enabling fast information recording of ∼225 kB/s and high-density data storage of ∼500 TB/disk (Figure 1C).

Despite all these advances, nanograting-based 5D optical data storage technology is still in its early stages and is far from being ready for practical large-scale applications. Many key issues are still waiting to be resolved, such as the fact that recording and reading speeds are still limited (Kazansky et al., 2020), and the resolution of azimuth (Fedotov et al., 2021b) and optical delays needs to be improved (Wang et al., 2022). But what is certain is that nanogratings will open up new avenues for high-performance and high-density data storage in the future.

### Optical components

Nanograting structures, due to their birefringent properties, are often used as basic elements of polarization conversion devices and geometric phase devices. By spatially distributed orientation and other parameters, devices are prepared on different materials and exhibit integration, miniaturization, and excellent performance.

Bricchi et al. (2002) exported the first nanograting-based diffractive element, a Fresnel band sheet, produced by direct writing of a femtosecond laser in quartz glass. Following this work, additional polarization-sensitive optical elements have been demonstrated, including optical attenuators (Zhang et al., 2013), polarization converters (Gertus and Kazansky, 2014), and optical vortex converters (Beresna et al., 2011).

Zhang et al. (2019b) prepared line arrays of nano-grating structures on circular glass with a diameter of 2 mm using femtosecond laser direct writing, and prepared near-infrared attenuators with high attenuation ratios based on polarization-dependent birefringence properties. The performance of the attenuator was tested by rubbing with a probe light source, and the zero-level diffraction signal intensity was received with a power meter, and the attenuator was found to satisfy a wide range of wavelength attenuation with symmetry.

The formation of uniform nano-grating is the key to reducing the optical loss in the birefringent modification of quartz glass. Therefore, it is very important to study the low loss of nanogratings. Sakakura et al. (2020) prepared nanopores extending along the vertical polarization direction by direct writing in quartz with a femtosecond laser of 300 fs pulse width and 200 kHz repetition frequency. Then, using this anisotropic nanoporous silica, nanograting structures were prepared by femtosecond laser induction. By regulating the birefringence of the irradiated region, the propagation direction and focus scattering of the incident circularly polarized light can be controlled. The birefringent low-loss modification of the material can be achieved, and the scattering loss is very low. The transmittance reaches 99% in the visible range and more than 90% in the ultraviolet spectrum below 330 nm. As shown in Figure 1D, GP prisms, GP lenses and polarization converters with ultra-high transmittance in a wide spectral range have been prepared using this method (Sakakura et al., 2020).

In addition, there is a stress field around the nanograting, which will also produce the stress birefringence effect. Compared with the uniform nanograting birefringence, it can better reduce the optical loss. In 2020, Tian et al. (2020) prepared two kinds of birefringent half waveplate using nano-grating birefringence and stress birefringence silicon glass respectively. By comparison, it is found that the optical loss of half waveplate based on nanogratings is higher because of the scattering of porous layer. On the contrary, the mm-sized half waveplate made based on the stress birefringence effect not only has low optical loss, but also can work stably at 1,000°C. The advantages of stress birefringence are well demonstrated.

In summary, nanogratings play a significant role in the preparation of optical devices, and reducing the birefringence loss of nanogratings to improve device performance and applying nanogratings to optical fiber systems will be a popular research direction in the future.

## Conclusion and outlook

The preparation and application of transparent material nanogratings based on 5D optical storage and polarized optical elements have taken a stride forward. By tuning the birefringence and eras-ability of the nano-grating structure, higher density and longer lifetime data storage can be achieved. And by regulating the generation of uniform nanogratings to reduce the birefringence loss rate, it is possible to obtain better performance of polarized optical devices. In summary, the tuning of femtosecond laser parameters and the selection of transparent material composition can be adjusted to obtain grating structures that are more suitable for practical applications. More research is needed in the future to further the study of femtosecond laser-induced nano-gratings inside transparent materials.
